# Expression of Mutant or Cytosolic PrP in Transgenic Mice and Cells Is Not Associated with Endoplasmic Reticulum Stress or Proteasome Dysfunction

**DOI:** 10.1371/journal.pone.0019339

**Published:** 2011-04-29

**Authors:** Elena Quaglio, Elena Restelli, Anna Garofoli, Sara Dossena, Ada De Luigi, Luigina Tagliavacca, Daniele Imperiale, Antonio Migheli, Mario Salmona, Roberto Sitia, Gianluigi Forloni, Roberto Chiesa

**Affiliations:** 1 Dulbecco Telethon Institute, Milan, Italy; 2 Department of Neuroscience, Mario Negri Institute for Pharmacological Research, Milan, Italy; 3 Department of Biochemistry and Molecular Pharmacology, Mario Negri Institute for Pharmacological Research, Milan, Italy; 4 Division of Genetics and Cell Biology, San Raffaele Scientific Institute and Università Vita-Salute San Raffaele, Milan, Italy; 5 Neurology Unit, Human Prion Diseases Center D.O.M.P., Maria Vittoria Hospital, Torino, Italy; University of Melbourne, Australia

## Abstract

The cellular pathways activated by mutant prion protein (PrP) in genetic prion diseases, ultimately leading to neuronal dysfunction and degeneration, are not known. Several mutant PrPs misfold in the early secretory pathway and reside longer in the endoplasmic reticulum (ER) possibly stimulating ER stress-related pathogenic mechanisms. To investigate whether mutant PrP induced maladaptive responses, we checked key elements of the unfolded protein response (UPR) in transgenic mice, primary neurons and transfected cells expressing two different mutant PrPs. Because ER stress favors the formation of untranslocated PrP that might aggregate in the cytosol and impair proteasome function, we also measured the activity of the ubiquitin proteasome system (UPS). Molecular, biochemical and immunohistochemical analyses found no increase in the expression of UPR-regulated genes, such as *Grp78/Bip*, *CHOP/GADD153*, or ER stress-dependent splicing of the mRNA encoding the X-box-binding protein 1. No alterations in UPS activity were detected in mutant mouse brains and primary neurons using the Ub^G76V^-GFP reporter and a new fluorogenic peptide for monitoring proteasomal proteolytic activity *in vivo*. Finally, there was no loss of proteasome function in neurons in which endogenous PrP was forced to accumulate in the cytosol by inhibiting cotranslational translocation. These results indicate that neither ER stress, nor perturbation of proteasome activity plays a major pathogenic role in prion diseases.

## Introduction

Prion diseases are fatal degenerative disorders of the central nervous system (CNS) that can arise sporadically, be genetically inherited, or acquired through infection [1], [2]. The key pathogenic event is conversion of the cellular prion protein (PrP^C^) into a self-propagating aberrant conformer (PrP^Sc^) [1], which induces neurodegeneration through an unknown mechanism [3]. PrP^C^ is a glycosylphosphatidylinositol (GPI) membrane-anchored glycoprotein of uncertain function, mainly expressed by neurons in the CNS [4]. Like most membrane-associated proteins, PrP^C^ is cotranslationally translocated into the endoplasmic reticulum (ER), where it undergoes oxidative folding and facultative N-linked glycosylation. After transit in the Golgi, PrP^C^ is delivered to the cell surface, where it resides in lipid rafts. Cell surface PrP^C^ can be released into the extracellular space, or internalized to an endosomal compartment, from which it is either recycled to the plasma membrane or diverted to lysosomes for degradation [5].

Mutations in the gene encoding PrP^C^ are responsible for genetic prion diseases, which include familial Creutzfeldt-Jakob disease (fCJD), Gerstmann-Sträussler-Scheinker (GSS) syndrome and fatal familial insomnia (FFI) [6], [7]. These diseases are inherited in an autosomal dominant fashion and are thought to be due to a toxic effect of misfolded mutant PrP molecules [3]. Tg(PG14) mice expressing the mouse PrP homologue of a 72-amino acid insertion associated with a mixed CJD-GSS phenotype develop a progressive neurological illness characterized by ataxia and massive apoptosis of cerebellar granule neurons [8], [9]. Tg(CJD) mice carrying the mouse PrP homologue of the D178N/V129 PrP mutation (moPrP D177N/V128) develop key features of fCJD, including cognitive, motor and neurophysiological abnormalities [10]. Tg(PG14) and Tg(CJD) mice synthesize in their brains misfolded forms of mutant PrP with biochemical properties reminiscent of PrP^Sc^, including insolubility in non-denaturing detergents, and resistance to mild proteinase-K (PK) digestion [8], [10]. Analysis of PG14 and D177N/V128 metabolism and localization in cultured neurons showed that the biosynthetic maturation of these mutants in the ER is delayed, and they accumulate abnormally in this organelle [10], [11], [12]. This suggests that perturbation of ER homeostasis due to accumulation of misfolded PrP may play a pathogenic role.

The ER has a robust quality control system that prevents misfolded and mismodified proteins from exiting the ER and being transported through the secretory pathway. This system includes chaperones and folding catalysts that form a matrix on which newly synthesized proteins attain their final conformation [13]. When protein misfolding occurs and unfolded proteins accumulate and aggregate in the ER, cells experience “ER stress” and activate an adaptive signal transduction pathway, known as the unfolded protein response (UPR). The UPR enhances the folding capacities in the ER, improves protein disposal through ER-associated degradation (ERAD), and reduces the rate of protein synthesis and translocation into the ER lumen [14].

The UPR signals through three distinct ER stress sensors on the ER membrane [15]. One is IRE1α (inositol-requiring transmembrane kinase and endonuclease), a type I ER transmembrane protein whose cytoplasmic region carries protein kinase and endoribonuclease domains. In ER-stressed cells IRE1α multimerizes and autophosphorylates, setting in motion its RNAse activity. Activated IRE1α initiates the unconventional splicing of the mRNA encoding the transcriptional factor X-box-binding protein 1 (XBP1) to produce sXBP1, a more stable form of XBP1 with a potent transactivator domain that enhances transcription of genes involved in protein folding, secretion and ERAD [16].

Another ER stress sensor is the activating transcription factor 6 (ATF6) [17]. This is a type II ER transmembrane protein whose cytosolic domain contains a bZIP transcriptional factor. When protein misfolding and accumulation occur, ATF6 is transported to the Golgi where it is processed within the transmembrane domain to release the cytosolic domain, which translocates to the nucleus and induces expression of the ER chaperone Grp78/BiP, and XBP1. Therefore, the IRE1α and ATF6 signaling pathways merge through regulation of XBP1 activity [18].

The third sensor is the ER-associated kinase PERK (double-stranded RNA-activated protein kinase-like ER kinase), which phosphorylates the α subunit of eukaryotic translation initiation factor 2 (eIF2α). This inhibits protein translation, reducing the overload of misfolded proteins. This pathway also activates ATF4, a transcription factor that induces expression of CHOP/GADD153, which can trigger apoptosis when cells unable to handle the unfolded protein load become irreversibly damaged. Thus, persistent ER stress eventually leads to cell death.

In this study we explored the role of ER stress in genetic prion diseases by measuring the levels of key elements of the UPR in brain tissue and primary neurons from Tg(PG14) and Tg(CJD) mice, and in transfected cells expressing mutant PrP under the control of a tetracycline-inducible promoter. Because ER stress favors formation of a cytosolic pool of untranslocated PrP [19], [20] that might aggregate and inhibit proteasome activity [21], we also examined the function of the ubiquitin-proteasome system (UPS) in the transgenic models, and in neurons in which PrP^C^ was forced to accumulate in the cytosol by interference with the correct insertion of the PrP signal peptide into the translocon.

## Results

### ER Stress Markers in Transgenic Mice and Primary Neuronal Cultures

To test whether mutant PrP expression triggered toxic response pathways in the ER, we analyzed the splicing of XBP1 mRNA transcripts and the levels of Grp78/BiP, Grp58/Erp57 and CHOP/GADD153 in the brains of Tg(PG14) and Tg(CJD) mice at different stages of their neurological illness. Tg(PG14) mice develop a slowly progressive neurological syndrome characterized neuropathologically by synaptic loss in the molecular layer of the cerebellum and massive apoptosis of cerebellar granule neurons [8], [9], [22]. They develop ataxia, kyphosis and foot clasp reflex at ∼240 days of age and die at ∼450 days [8], [9]. Tg(CJD) mice develop alterations of spatial working memory between 200 and 320 days of age, electroencephalographic abnormalities at ≥360 days, and a motor syndrome involving alterations of motor coordination and body posture that is first seen at ∼450 days and increases in severity as the mice age [10]. Consistent neuropathological findings in Tg(CJD) mice are loss of calretinin-positive neurons in the hippocampus and neocortex, and ER swelling with accumulation of mutant PrP in cerebellar granule neurons [10].

ER stress markers were analyzed in a total of 23 Tg(PG14) mice aged 23, 24, 36, 102, 120, 124, 131, 146, 171, 172, 189, 210, 214, 226, 240, 241, 296, 364, 372, 377, 404, 405 and 409 days, and five Tg(CJD) mice at 81, 338, 411, 421 and 814 days. RNA and proteins were extracted from the whole brain or specific brain regions showing pathological changes (the cerebellum in Tg(PG14) mice, and the cerebral cortex, hippocampus and cerebellum in Tg(CJD) mice). Non-Tg littermates and age-matched Tg(WT) mice expressing wild-type PrP [8] were used as controls.

**Figure 1:**
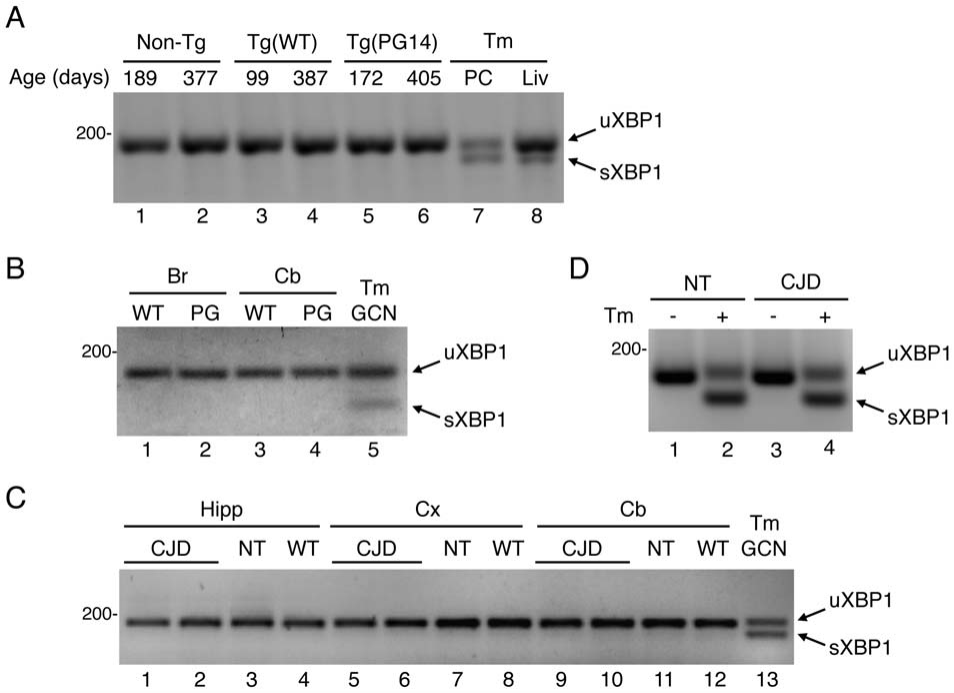
Analysis of XBP1 splicing in mutant PrP transgenic mice and primary neurons. (A) Total RNA was extracted from the whole brain of two male Non-Tg (lanes 1 and 2), two female Tg(WT) (lane 3 and 4), one presymptomatic female (lane 5) and one clinically ill male (lane 6) Tg(PG14) mice of the ages indicated, and analyzed by RT-PCR. XBP1 splicing was determined from the appearance of rapidly migrating spliced XBP1 in tunicamycin (Tm)-treated PC12 cells (PC, lane 7) or in the liver of a Non-Tg mouse intraperitoneally injected with tunicamycin (1 µg per gram of body weight) and analyzed 96 h later (Liv, lane 8). The arrows point to unspliced (uXBP1) and spliced (sXBP1) transcripts. (B) XBP1 splicing was analyzed in whole brain (Br) or cerebellum (Cb) of a male Tg(WT) (WT, lanes 1 and 3) and a female Tg(PG14) mouse with early signs of neurological disease (PG, lanes 2 and 4), aged 260 and 240 days respectively. Cultured cerebellar granule neurons from C57BL/6J mice treated with Tm were used as positive control (CGN, lane 5). (C) Total RNA was extracted from the hippocampus (Hipp), cerebral cortex (Cx) and cerebellum (Cb) of a 411-day-old female (lanes 1, 5 and 9), and a 421-day-old male (lanes 2, 6 and 10) Tg(CJD-A21) mouse (CJD), a 448-day-old female Non-Tg mouse (NT, lanes 3, 7 and 11) and a 341-day-old female Tg(WT) mouse (WT, lanes 4, 8 and 12). Tm-treated cerebellar granule neurons (CGN) were used as control (lane 13). (D) CGN from Non-Tg (NT) and Tg(CJD) mice were cultured for 7 days, and either left untreated (−) or treated (+) with 5 µg/ml Tm for 8 h before analysis of XBP1 splicing. This result is representative of two independent experiments. Size markers are given in base-pairs.

**Figure 2:**
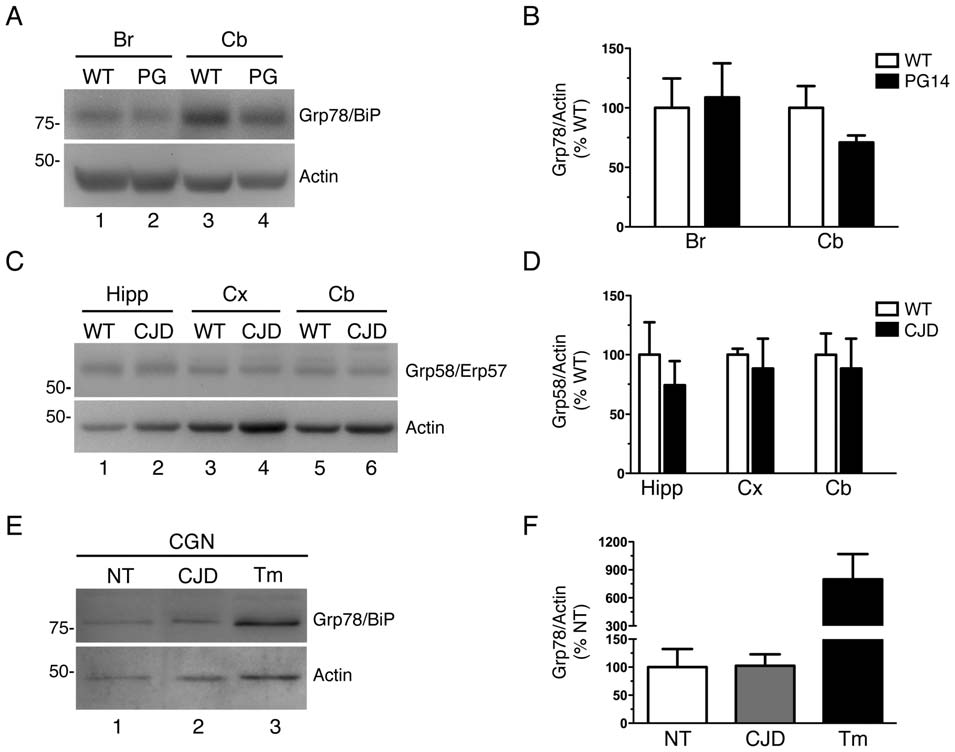
The ER stress-regulated chaperones Grp78/BiP and Grp58/Erp57 are not induced in the brain and primary neurons from mutant PrP mice. (A) Protein extracts (20 µg) from the whole brain (Br) or cerebellum (Cb) of a 358-day-old male Tg(WT) and a terminally ill 363-day-old male Tg(PG14) mouse were analyzed by Western blot with anti-KDEL and anti-actin antibodies. The anti-KDEL antibody detects a band at ∼78 kDa corresponding to Grp78/BiP. Molecular mass markers are in kilodaltons. (B) Grp78/BiP was quantified by densitometic analysis of Western blots like the one shown in A, normalized for the level of actin, and expressed as a percentage of the level in Tg(WT) mice. Each bar indicates the mean ± SEM of three animals. (C) Protein extracts (20 µg) from the hippocampus (Hipp), cerebral cortex (Cx) and cerebellum (Cb) of a 323-day-old male Tg(WT) and a 338-day-old male Tg(CJD) mouse were analyzed by Western blot with anti-Grp58/Erp57 and anti-actin antibodies. (D) Grp58/Erp57 level was quantified by densitrometic analysis of Western blots, as shown in C, normalized for the level of actin, and expressed as a percentage of the level in Tg(WT) mice. Each bar indicates the mean ± SEM of three animals. (E) Protein extracts of cerebellar granule neurons (CGN) from Non-Tg (NT) and Tg(CJD) mice were immunoblotted with anti-KDEL and anti-actin antibodies. CGN from C57BL/6 mice were treated with tunicamycin (Tm) as positive control. (F) Grp78/BiP level was quantified by densitrometic analysis of Western blots as the one shown in E, normalized for the level of actin, and expressed as a percentage of the level in Non-Tg mice. Each bar represents the mean ± SEM of four independent cultures.

XBP1 splicing was readily detected in the liver of mice and in cells treated with tunicamycin, which induces ER stress by perturbing the folding efficiency in the ER, whereas no splicing was observed in the whole brain or dissected brain regions of the mutant mice at any stage of their disease (Fig. 1A-C and data not shown). There was no increase in the levels of Grp78/BiP or CHOP/GADD153 transcripts, assessed by Northern blot (not shown), or Grp58/Erp57 and Grp78/BiP proteins by Western blot of brain extracts (Fig. 2A–D).

**Figure 3:**
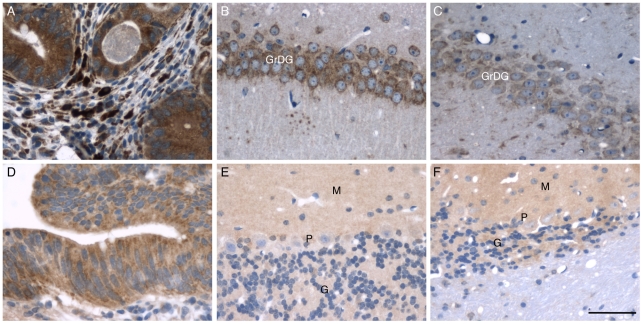
Mutant PrP transgenic mice show no increase in Grp78/BiP and CHOP/GADD153 immunoreactivity in the brain. (A–C) Immunohistochemical detection of Grp78/BiP in human colon adenocarcinoma (A), and in the hippocampus of a 504-day-old female Tg(WT) (B) and a 751-day-old female Tg(CJD) mouse with terminal disease (C). (D–F) Immunohistochemical detection of CHOP/GADD153 in human colon adenocarcinoma (D), and in the cerebellar cortex of the Tg(WT) mouse shown in B (E) and a 367-day-old male Tg(PG14) mouse with terminal disease (F). Note the reduced number of cerebellar granule neurons in the Tg(PG14) mouse. M, molecular layer; P, Purkinje cell layer; G, granule cell layer; GrDG, granular zone of the dentate gyrus. Scale bar  = 50 µm.

In a final set of experiments, we used immunohistochemical techniques to visualize UPR activation in brain sections of four Tg(PG14) mice aged 145, 180 and 367 (two mice) days, and two Tg(CJD) mice aged 542 and 751 days. As a positive control for the staining, Grp78/BiP and CHOP/GADD153 were readily detected in colon adenocarcinoma biopsies (Fig. 3A and D) [23], [24]. No increase in immunoreactivity was seen in the brains of Tg(PG14) and Tg(CJD) mice compared to age-matched Non-Tg and Tg(WT) controls, not even in the regions undergoing pathological changes (Fig. 3C and F).

The fact that we detected no proximal UPR signaling in the brain might be due to transient and/or not synchronized UPR activation. Therefore we analyzed UPR markers in primary cultures of CGN from Tg(CJD) mice, which show dramatic ER swelling associated with retention of mutant PrP [10]. No XBP-1 splicing was observed in CGN from the mutant mice, unless cells were treated with tunicamycin (Fig. 1D). There was also no difference in Grp78/BiP protein levels between control and mutant CGN (Fig. 2E and F).

### ER Stress in PC12 Cells Expressing Mutant PrP Under the Control of an Inducible Promoter and in Transiently Transfected HEK-293 cells

To exclude that cells unresponsive/resistant to the effect of the mutant protein were selected in culture, we developed lines of transfected cells in which PrP expression was activated after clonal selection. We used pheochromocytoma PC12 cells, which respond to nerve growth factor (NGF) by stopping division and developing the morphological, electrophysiological and neurochemical properties of neurons [25]. PC12 cells constitutively expressing the reverse tetracycline-controlled transactivator (PC12 Tet-on) [26] were transfected with an expression vector in which the PrP cDNA was under the control of the tetracycline-responsive element (TRE). To prevent unregulated PrP expression in the absence of inducer doxycycline (dox), cells were co-transfected with a plasmid encoding the tet-control transcriptional silencer which blocks transcription of genes under control of the TRE in the absence of dox. We generated PC12 Tet-on cell lines expressing PG14 and D177N/V128, as well as D177N/M128 PrP whose human homolog is associated with FFI. As controls, we produced lines expressing mouse wild-type PrP (M128), and M128V PrP to model the non-pathogenic V129 polymorphic variant of human PrP.

**Figure 4:**
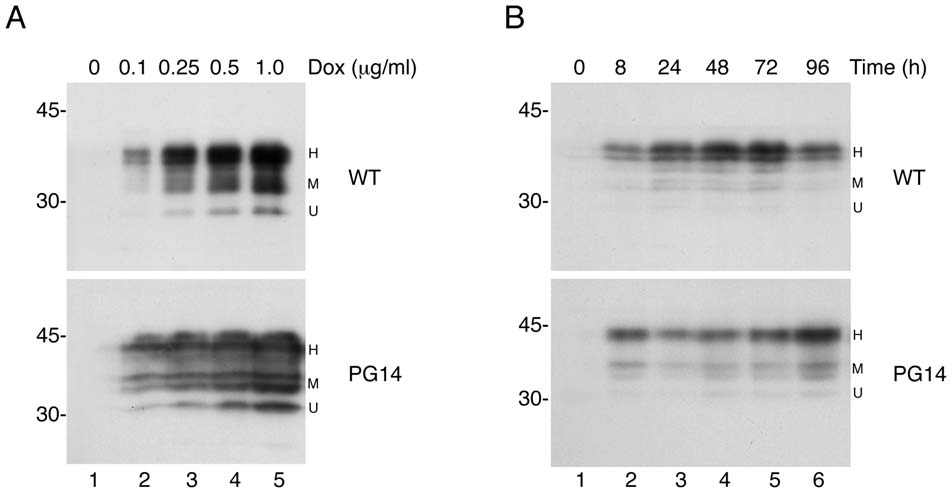
Regulation of PrP expression in PC12 Tet-on cells. (A) PC12 Tet-on cells transfected with WT (top panel) or PG14 (lower panel) PrP were treated with the doses indicated of doxycycline (dox) and lysed after 24 h. Lysates corresponding to 50 µg of proteins were analyzed by Western blot using the 3F4 antibody. (B) Cells were induced with 0.25 µg/ml dox for the indicated times and analyzed as in A. Molecular mass markers are in kDa. Similar results were obtained with clones of PC12 cells transfected with M128V, D177N/M128 or D177N/V128 PrPs (not shown). U, unglycosylated; M, monoglycosylated; H, highly glycosylated. Note that PG14 PrP has a higher molecular mass because of the 72-amino acid insertion.

PC12 Tet-on cells had tightly regulated expression of PrP, which was undetectable in the absence of dox, and which increased progressively with increasing dox concentrations (Fig. 4A). PrP expression was detected after 8 h of induction, and lasted at least 96 h (Fig. 4B). Most PC12 clones displayed maximal transgenic PrP expression (approximately double endogenous PrP) in response to 1–2 µg/ml dox (not shown).

**Figure 5:**
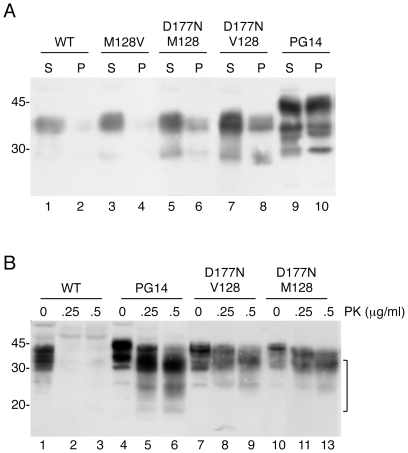
Mutant PrPs acquire abnormal biochemical properties in PC12 Tet-on cells. (A) Cells transfected with WT, M128V, D177N/M128, D177N/V128 or PG14 PrP were induced with 1 µg/ml dox for 24 h before lysis. 100 µg of protein extract was ultracentrifuged for 40 min at 186,000 x *g* to separate the soluble (S) and insoluble (P) protein fractions, and PrP was analyzed by Western blot using antibody 3F4. (B) Cells transfected with WT or mutant PrPs were induced with 1 µg/ml dox for 24 h before lysis. 300 µg of protein extract was digested with the indicated concentrations of PK and analyzed by Western blot using antibody 3F4. The bracket indicates the bands corresponding to PK-resistant PrP.

**Figure 6:**
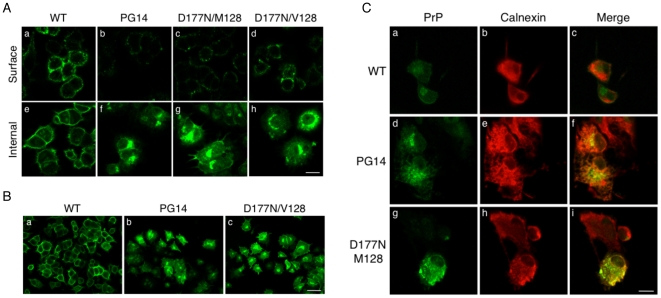
Mutant PrPs levels are lower on the surface of PC12 cells and co-localize with an ER marker. (A) PC12 Tet-on cells transfected with WT, PG14, D177N/M128 or D177N/V128 PrP were induced with 1 µg/ml dox for 24 h. Cells were incubated with 3F4 antibody without permeabilization to detect PrP on the cell surface (panels a–d) or fixed and permeabilized before incubation with 3F4 to visualize intracellular PrP too (panels e–h). Scale bar  = 10 µm. (B) Low-magnification images of PC12 Tet-on cells transfected with WT, PG14 and D177N/V128 after permeabilization and immunofluorescence staining of PrP. Scale bar  = 20 µm. D177N/M128 PrP gave similar results (not shown). (C) PC12 Tet-on cells transfected with WT, PG14, D177N/M128 PrPs were differentiated with 100 ng/ml NGF, and PrP expression was induced with 1 µg/ml dox for 24 h. Cells were fixed, permeabilized and stained with mouse monoclonal anti-PrP antibody 3F4 (panels a, d and g) and rabbit anti-calnexin antibody (panels b, e and h) followed by Alexa 488(green)-conjugated anti-mouse and Alexa 546(red)-conjugated anti-rabbit secondary antibodies. Merged images are shown in panels c, f and i. Scale bar  = 10 µm. D177N/V128 PrP gave similar results (not shown).

Mutant PrPs were insoluble in non-denaturing detergents (Fig. 5A; percentages of PrP in the pellet were: 68±12 for PG14; 44±8.2 for D177N/M128; 47±10.5 for D177N/V128; n = 5), and were weakly protease-resistant, in contrast to the solubility and protease-sensitivity of WT and M128V PrPs (Fig 5A and B, and data not shown). Consistent with previous observations [27], neuronal differentiation of PC12 cells on exposure to NGF did not modify the proportion of detergent-insoluble PrP or its level of protease resistance (not shown). The mutant PrPs expressed in PC12 Tet-on cells were found at lower levels on the cell surface, and had an intense intracellular distribution (Fig. 6A and B), which colocalized in part with the ER marker calnexin (Fig. 6C). Thus PG14 and D177N PrPs misfolded and were partially retained in the ER of PC12 Tet-on cells, confirming similar observations in primary neurons [10], [12], and validating this cell model for investigating the effect of acute mutant PrP expression on ER homeostasis.

**Figure 7:**
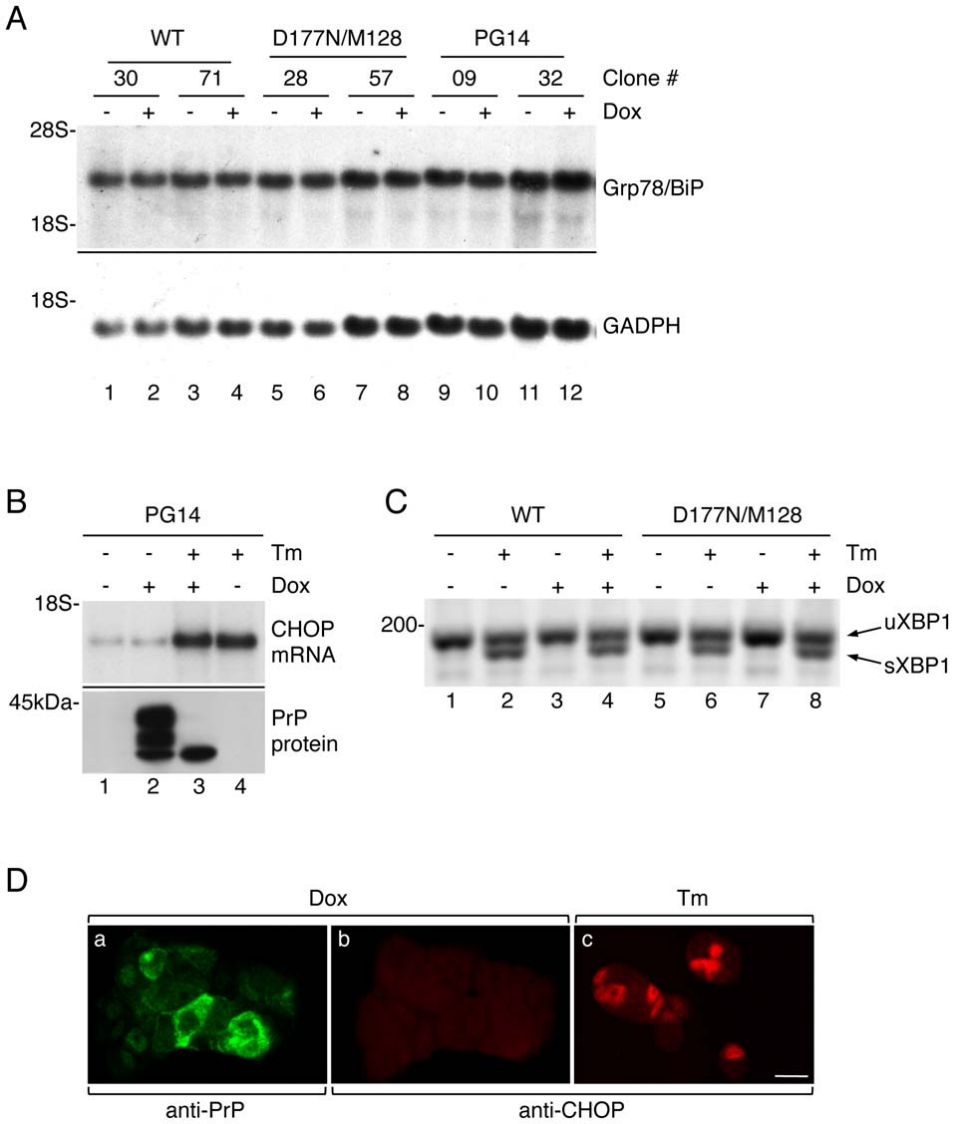
Mutant PrP expression does not trigger ER stress response in PC12 Tet-on cells. (A) Two clones of PC12 Tet-on cells transfected with WT (lanes 1–4), D177N/M128 (lanes 5–8) or PG14 PrP (lanes 9–12) were left untreated (−) or induced for 24 h with 1 µg/ml dox (+); 20 µg of total RNA was analyzed by Northern blot using Grp78/BiP-(top panel) and GAPDH-(lower panel) specific probes. The D177N/V128 mutant did not behave differently from the D177N/M128 and so is not included in this figure. (B) Cells transfected with PG14 PrP were left untreated (−) or treated (+) for 24 h with 1 µg/ml dox, with or without 1 µg/ml tunicamycin (Tm). Total RNA was extracted for Northern blot analysis with a CHOP-specific probe (top panel). Sister cells were lysed for Western blot analysis with antibody 3F4 to verify induction of PrP expression and the effectiveness of tunicamycin, demonstrated by the appearance of a single band corresponding to unglycosylated PrP (lower panel). Clones transfected with D177N/M128 or D177N/V128 PrP gave the same result so are not included in this figure. (C) Cells transfected with WT or D177N/M128 PrP were left untreated (−) or treated (+) for 24 h with 1 µg/ml dox, with or without 1 µg/ml Tm, or with Tm but not dox. Total RNA was extracted and XBP1 splicing was analyzed by RT-PCR. Each product of amplification was separated on 2.5% agarose gel. Spliced forms were detected only in samples treated with tunicamycin. Clones transfected with M128V, PG14 or D177N/V128 PrP gave the same result so are not included in this picture. (D) Cells transfected with PG14 PrP were treated with 1 µg/ml dox for 24 h (panels a and b) or with tunicamycin (panel c), and analyzed by immunofluorescence with mouse monoclonal anti-PrP antibody 3F4 (panel a) and rabbit anti-CHOP antibody (panels b and c) followed by Alexa 488(green)-conjugated anti-mouse and Alexa 546(red)-conjugated anti-rabbit secondary antibodies. Panels a and b show the same field. Clones transfected with D177N/M128 or D177N/V128 PrP gave the same result. Scale bar  = 10 µm.

The levels of Grp78/BiP and CHOP/GADD153 mRNAs were analyzed in PC12 Tet-on cells before and after induction with 1 µg/ml dox for 24 h. PrP expression did not increase the amount of these transcripts (Fig. 7A and B). There was also no difference in Grp78/BiP protein levels between control and mutant cells (not shown). IRE1α-dependent splicing of XBP1 mRNA was then assessed in cells treated with tunicamycin and/or dox. The spliced form of XBP1 was readily detectable after tunicamycin, but not in cells exposed only to dox (Fig. 7C). The same analysis on cells treated with dox for 96 h showed no evidence of ER stress (not shown).

It was possible that only a limited number of cells accumulated sufficient amounts of mutant PrP in the ER to activate the ER stress response. If this were the case, analysis of total RNA extracted from whole cultures could contain low levels of Grp78/BiP, CHOP/GADD153 and XBP1 transcripts. To directly test whether UPR markers were activated in cells accumulating high PrP levels, mutant PrP-transfected cells were treated with dox or tunicamycin then immunostained with anti-PrP and anti-CHOP/GADD153 antibodies. There was no activation of CHOP/GADD153 in cells accumulating high intracellular PrP levels (Fig. 7D, panels a and b); as expected, cells treated with tunicamycin showed intense staining, indicative of CHOP/GADD153 induction (Fig. 7D, panel c).

Finally, we analyzed ER stress markers in a different cell model of acute mutant PrP expression. HEK-293 cells were transfected with an empty pcDNA3.1 plasmid or with plasmids encoding WT, PG14, D177N/M128 or D177N/V128 PrP. As positive controls cells were transfected with a plasmid encoding murine Ig-µ-chains, which in the absence of Ig-L chains accumulate in the ER inducing a robust UPR ([28], [29] and T. Anelli, R. Ronzoni and R.S., unpublished results), or were treated with tunicamycin. Cells were lysed 24 h after transfection to monitor PrP and µ-chain expression and XBP1 mRNA splicing. PrP and µ-chains were efficiently expressed in HEK-293 cells (Fig. S1A and B). XBP1 mRNA splicing was readily detected in µ-chain expressing cells or after treatment with tunicamycin, but not in mutant PrP-expressing cells (Fig. S1C). Thus, although HEK-293 cells respond normally to misfolded protein accumulation in the ER by up-regulating the UPR system, mutant PrPs did not evoke this response.

### Mutant PrP Mice or Neurons Accumulating Cytosolic PrP Show no Functional Impairment of Ubiquitin-dependent Proteasomal Degradation

It has been suggested that misfolded PrP might be recognized as abnormal by the ER quality control machinery and retrogradely translocated into the cytosol for proteasomal degradation (ER-associated degradation, ERAD, pathway) [30], [31]. In Tg(PG14) mice, mutant PrP accumulates continuously as the animals age and granule neurons in the cerebellum die [9]. To test whether accumulation of mutant PrP in Tg(PG14) mice was associated with reduced proteasome function, we generated double-transgenic mice co-expressing PG14 PrP and a green fluorescent protein (GFP) reporter substrate of the UPS (Ub^G76V^-GFP) [32]. The Ub^G76V^-GFP transgene is under the transcriptional control of the β-actin promoter, which drives constitutive expression in all organs. Under physiological conditions the N-terminal ubiquitin moiety of Ub^G76V^-GFP is recognized as a degradation signal and poly-ubiquitinated, leading to constitutive proteosomal degradation of GFP. An alteration of the UPS therefore results in accumulation of Ub^G76V^-GFP. Expression of this reporter *in vivo* has been used to assess the UPS function in prion-infected mice [21], and in transgenic mouse models of polyglutamine disease and amyotrophic lateral sclerosis [33], [34], [35].

Tg(PG14) mice were crossed with two independent lines of Ub^G76V^-GFP mice (Ub^G76V^-GFP1 and Ub^G76V^-GFP2), which have different basal levels of reporter expression, to produce Tg(PG14^+/-^)/Ub^G76V^-GFP^+/−^ mice, and the Tg(PG14^−/−^)/Ub^G76V^-GFP^+/−^ and Tg(PG14^+/−^)/Ub^G76V^-GFP^−/−^ controls. As an additional control, we generated Tg(WT^+/−^)/Ub^G76V^-GFP^+/−^ mice, expressing transgenically-encoded wild-type PrP. There were no differences in the onset and progression of disease between Tg(PG14) mice that expressed or did not express Ub^G76V^-GFP, judging by a set of objective criteria [8].

**Figure 8:**
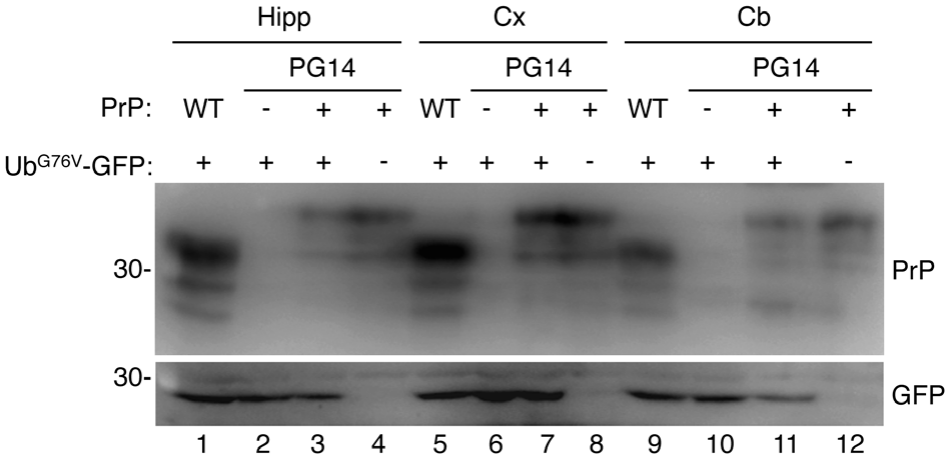
Tg(PG14) mice do not accumulate Ub^G76V^-GFP in their brains. The hippocampus (Hipp), cerebral cortex (Cx) and cerebellum (Cb) were dissected from a 181-day-old Tg(WT^+/−^)/Ub^G76V^-GFP1^+/−^ (lanes 1, 5 and 9), a 187-day-old Tg(PG14^−/−^)/Ub^G76V^-GFP1^+/−^ (lanes 2, 6 and 10), a Tg(PG14^+/−^)/Ub^G76V^-GFP1^+/−^ (lanes 3, 7 and 11), and a Tg(PG14^+/−^)/Ub^G76V^-GFP1^−/−^ (lanes 4, 8 and 12) mouse, and analyzed by Western blot (100 µg protein per lane) with antibody 3F4 which detects transgenic but not endogenous PrP (top panel), and anti-GFP antibody (lower panel).

**Figure 9:**
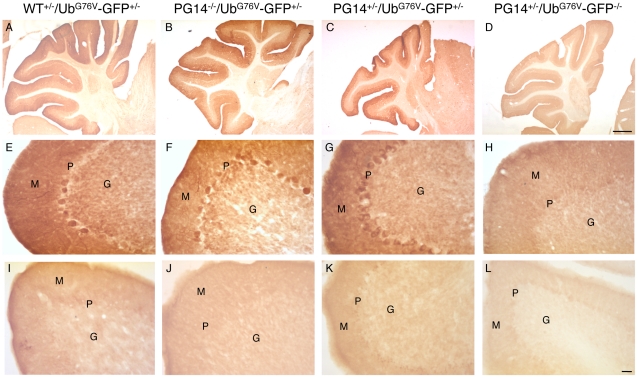
Cerebellar degeneration in Tg(PG14) mice is not associated with proteasome impairment. Sections from Tg(WT^+/−^)/Ub^G76V^-GFP1^+/−^ (A, E), Tg(PG14^−/−^)/Ub^G76V^-GFP1^+/−^ (B, F), Tg(PG14^+/−^)/Ub^G76V^-GFP1^+/−^ (C, G) and Tg(PG14^+/−^)/Ub^G76V^-GFP1^−/−^ mice (D, H); Tg(WT^+/−^)/Ub^G76V^-GFP2^+/−^ (I), Tg(PG14^−/−^)/Ub^G76V^-GFP2^+/−^ (J), Tg(PG14^+/−^)/Ub^G76V^-GFP2^+/−^ (K) and Tg(PG14^+/−^)/Ub^G76V^-GFP2^−/−^ (L) mice were stained with anti-GFP antibody. Mice were 292 (A–D), 150 (E–H), and 325 (I–L) days old. Aged PG14 PrP-expressing mice show marked atrophy of the cerebellum (C, D). Purkinje neurons of mice expressing the Ub^G76V^-GFP1 transgene (E–G) show specific GFP immunopositivity (not seen in the Ub^G76V^-GFP1^−/−^ mouse in panel H), irrespective of the PrP genotype. M, molecular layer; P, Purkinje cell layer; G, granule cell layer. Scale bar in D (applicable to A–D) = 500 µm; scale bar in L (applicable to E–L) = 200 µm.

Mice were culled at different stages of the Tg(PG14) illness [9], and their brains analyzed by Western blot and immunohistochemistry with anti-GFP antibodies. Western blot analysis was done in two Tg(PG14^+/−^)/Ub^G76V^-GFP1^+/−^ mice aged 91 days, and one each at 98, 150, 187, 248 and 293 days. Immunohistochemistry was done on two Tg(PG14^+/−^)/Ub^G76V^-GFP1^+/−^ aged 73 days, one each at 103, 150, 187, 277, 292, 313 and 333 days, and two Tg(PG14^+/−^)/Ub^G76V^-GFP2^+/−^ aged 141 days, two at 214 and one at 325 days. There was no increase in the level of the GFP reporter in Tg(PG14^+/−^)/Ub^G76V^-GFP^+/−^ mice compared to age-matched Tg(PG14^−/−^)/Ub^G76V^-GFP^+/−^ and Tg(WT^+/−^)/Ub^G76V^-GFP^+/−^ controls (Fig. 8 and 9). As previously reported [8], [9], mice expressing PG14 PrP showed marked atrophy of the cerebellum due to synaptic loss in the molecular layer and apoptosis of granule neurons (Fig. 9, compare panels C, D with A, B); this was not associated with any increase in immunoreactivity for GFP (Fig. 9, compare panel G with E and F; and K with I and J). Specific GFP-immunopositivity was detected in Purkinje neurons in the cerebellum of Tg(PG14^+/−^)/Ub^G76V^-GFP1^+/−^ mice expressing higher reporter levels (Fig. 9G). However, this was also detected in Tg(PG14^−/−^)/Ub^G76V^-GFP1^+/−^ and Tg(WT^+/−^)/Ub^G76V^-GFP1^+/−^ mice (Fig. 9E and F), and was therefore independent of mutant PrP expression.

**Figure 10:**
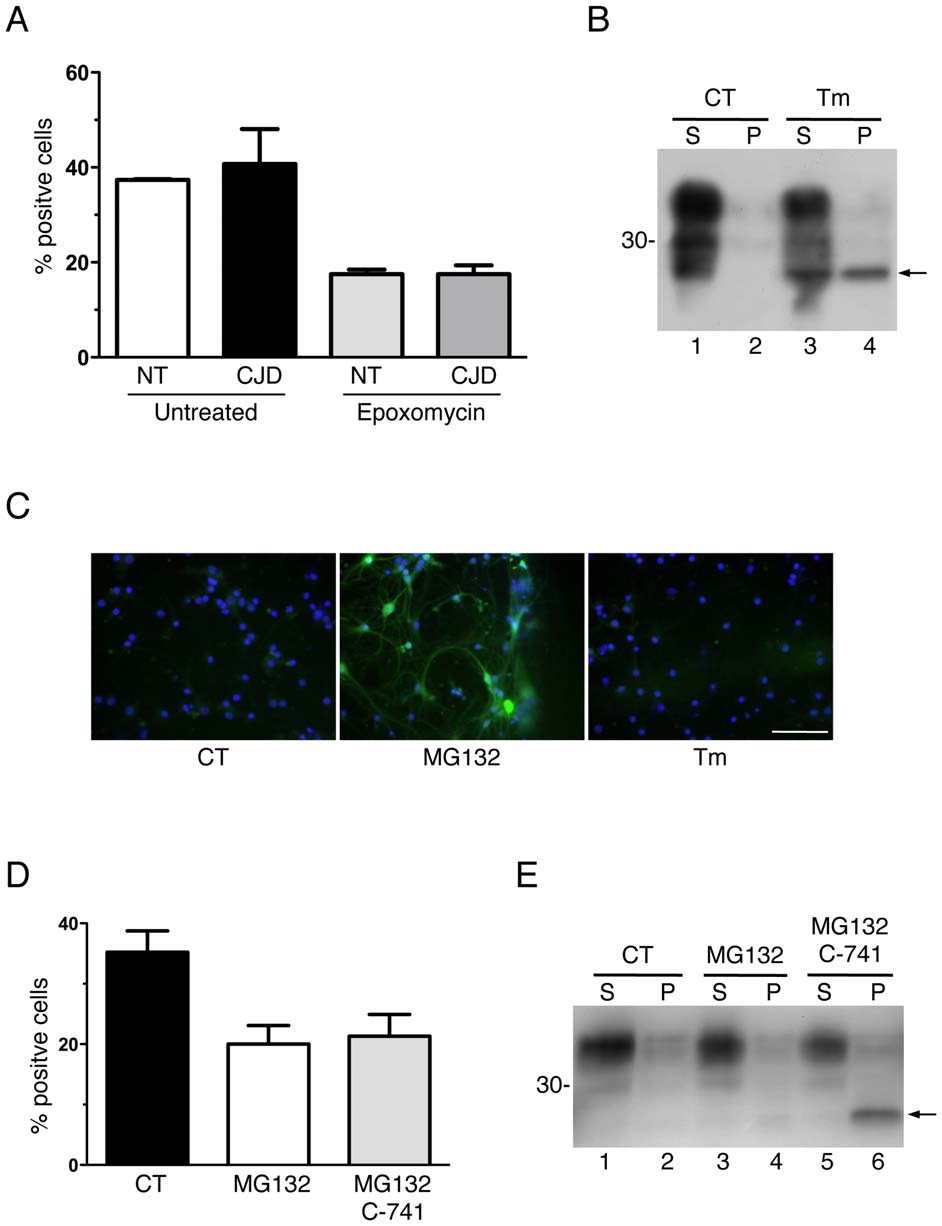
Expression of D177N PrP or accumulation of cytosolic PrP is not associated with reduced proteasome function. (A) Cultures of CGN from Tg(CJD) mice (CJD) and nontransgenic littermates (NT) were left untreated or exposed to 5 µM epoxomycin for 3 h, and incubated with 40 µM TED for 20 min to measure proteasome activity. Fluorescence-positive cells were quantified by optical microscopy. Data are the mean ± SEM of three independent experiments. (B) Cultures of hippocampal neurons from Ub^G76V^-GFP2^+/−^ mice were left untreated (CT) or exposed to 5 µg/ml tunicamycin (Tm) for 24 h. Cell were lysed and centrifuged at 186,000 x *g* for 40 min, and PrP in the supernatants (S) and pellet (P) was visualized by immunoblotting with antibody 12B2. The arrow indicates the band corresponding to untranslocated PrP. Molecular mass markers are in kilodaltons. (C) Hippocampal neurons from Ub^G76V^-GFP2^+/−^ mice were left untreated or exposed to 20 µM MG132 for 5 h or 5 µg/ml tunicamycin (Tm) for 24 h, then fixed and reacted with an anti-GFP antibody (green), and DAPI (blue) to stain the nuclei. Scale bar  = 100 µm. (D) Hippocampal neurons from C57BL/6J mice were left untreated or exposed to 10 µM C-741 for 18 h to inhibit PrP translocation; 20 µM MG132 was added during the last 6 h to induce accumulation of untranslocated PrP. Fluorescence-positive cells were quantified after 20 min incubation with TED. Data are the mean ± SEM of three independent experiments. (E) Untransocated PrP was visualized by immunoblotting as in B.

Next we assessed UPS activity in cultured transgenic neurons expressing D177N PrP. Primary CGN from Tg(CJD) mice and nontransgenic littermates were incubated with an internally quenched fluorogenic peptide, carrying a proteasome-specific cleavage motif fused to the TAT sequence and linked to the fluorophores EDANS and DABCYL (TED) [36]. The TED peptide penetrates cell membranes and is rapidly cleaved by the proteasomal chymotrypsin-like activity. Proteasomal cleavage leads to physical separation of the EDANS/DABCYL pair, abolishing intramolecular quenching, and EDANS fluorescence increases proportionally to the amount of substrate cleaved. Thus, proteasomal efficiency can be directly assessed by counting fluorescent cells by optical microscopy. The percentages of fluorescent neurons were comparable in Tg(CJD) and nontransgenic cultures, indicating that basal proteasome activity was not altered in cells expressing mutant PrP (Fig. 10A). The proteasome inhibitor epoxomycin had similar potency in Tg(CJD) and nontransgenic neurons (Fig. 10A).

ER stress activates a “pre-emptive” quality control system that aborts PrP translocation, allowing its proteasome-mediated degradation in the cytosol [19], [20]. Under chronic ER stress the proteasome may be overwhelmed [37], resulting in accumulation and aggregation of PrP in the cytosol [38]. Cytosolic PrP oligomers may inhibit proteasome activity [21], triggering a feedback loop leading to further PrP accumulation in the cytosol and cell death [39], [40]. We used two different approaches to test whether accumulation of untranslocated PrP aggregates was associated with low proteasome function. We treated hippocampal neurons from Ub^G76V^-GFP mice with tunicamycin to induce ER stress and untranslocated PrP (Fig. 10B, lane 4) [19], and monitored Ub^G76V^-GFP accumulation by live cell imaging and immunostaining with an anti-GFP antibody. Ub^G76V^-GFP was readily detectable in cells treated with the proteasome inhibitor MG132, but not with tunicamycin (Fig. 10C), indicating that cytosolic accumulation of untranslocated PrP did not inhibit proteasomal degradation of Ub^G76V^-GFP. Next, we assessed proteasome activity in cells treated with a cyclopeptolide (C-741) that inhibits PrP translocation [41], [42], producing a biologically active form of cytosolic PrP [42]. Hippocampal neurons from C57BL/6 mice were incubated with or without C-741 for 24 h. MG132 was added during the last 6 h of treatment to induce robust accumulation of untranslocated PrP in the cytosol of C-741-treated cells [42], and proteasome activity was measured using the TED peptide. We found no significant differences in proteasome function between cells treated with MG132 alone or together with C-741 (Fig. 10D), although the latter accumulated cytosolic PrP (Fig.10E, lane 6).

## Discussion

Secretory and membrane proteins are subject to a stringent surveillance mechanism that causes retention of misfolded forms in the ER preventing them from reaching the cell surface. Retained proteins can stimulate ER stress response pathways, such as the UPR, which transmits information to the cytosol and nucleus to improve protein folding capacity, inhibit protein synthesis and translocation, and favor misfolded protein catabolism through ERAD. Since PrP^Sc^ and other forms of pathogenic PrP are likely to be structurally distinct from PrP^C^, one might expect these molecules to be recognized as “abnormal” by the ER quality control machinery and trigger ER stress. Persistent ER stress may lead to apoptosis [14], a cell death modality that is often observed in experimental and natural prion diseases.

ER stress was proposed to play a key role in prion diseases since stress-regulated ER chaperons, such as Grp78/BiP and Grp58/Erp57, were induced in sporadic and variant CJD, and in bovine spongiform encephalopathy, and their up-regulation correlated with disease progression in prion-infected mice [38], [43], [44], [45], [46]. Treatment of neuroblastoma N2a cells with PrP^Sc^ purified from infected hamsters induced Grp78/BiP, Grp58/Erp57 and Grp94, and activated caspase-12, a protein involved in ER stress-induced apoptosis [44]. Similar changes were observed in cortical neurons treated with synthetic PrP peptide 106–126, an experimental tool used to model PrP^Sc^ neurotoxicity [47].

However, the cellular mechanism by which PrP^Sc^ could cause stress in the ER is not clear. PrP^Sc^ propagates itself by imprinting its aberrant conformation on endogenous PrP^C^, generating additional molecules of PrP^Sc^ in an autocatalytic reaction [48], [49]. This conversion occurs on the cell surface or within an endocytic compartment [50], [51], [52], so it is unlikely to be the proximate cause of stress in the ER. Exposure of N2a cells to purified PrP^Sc^ induced fast, sustained calcium release from ER stores [44], suggesting that perturbation of ER calcium homeostasis might be the actual trigger. Regardless of the precise mechanism by which PrP^Sc^ stimulates ER stress, the fact that genetic ablation of XBP1 or caspase-12 does not modify the onset or progression of symptoms in prion-infected mice [53], [54] indicates that this pathway is not essential for prion diseases acquired by infection.

ER stress, however, might play a pathogenic role in genetic prion diseases, in which the mutant PrP molecules misfold spontaneously in the ER lumen [55]. In the present study we measured the levels of several ER stress markers in transgenic mice and cells expressing PG14 and D177N PrPs. These mutant molecules accumulate in the neuronal secretory pathway [12], and their expression is associated with swelling of the ER cisternae ([10] and unpublished observations), a morphological alteration often associated with the UPR [56]. Unexpectedly, molecular biology, biochemical and immunohistochemical analyses of brain tissue and primary neurons from the mutant mice did not find evidence of an ER stress response. Analysis of transiently transfected HEK-293 cells or PC12 cells in which PrP expression was switched on only at the time of the experiment to rule out adaptation to mutant PrP gave similar results. Thus, chronic or acute mutant PrP expression was not associated with ER stress in transgenic models, consistent with evidence from genetic prion disease patients [57].

How might the lack of an ER stress response be explained despite demonstrable mutant PrP misfolding in this organelle? There is evidence that pathogenic mutations, including D178N, do not perturb the overall fold of PrP [58] but stabilize partially structured folding intermediates [59], which might elude the quality control system in the ER. We did in fact find that PG14 and D177N PrP were not diverted to ER-associated degradation [11], [12], but eventually trafficked to post-Golgi compartments [55] from which they are probably routed to the endo-lysosomal system for degradation [60].

Although ER stress does not appear to be a causative mechanism in prion diseases, perturbation of ER homeostasis might contribute to the pathogenesis. Treatment of N2a cells with several ER stressors caused formation of a PrP^C^ isoform that was more prone to PrP^Sc^-induced *in vitro* misfolding, suggesting that ER-damaged neurons may be more susceptible to prion replication [61]. Furthermore, ER stress could compromise UPS functionality [37] and damage cells by favoring formation of cytosolic PrP oligomers that might further inhibit proteasome function [21], [38], [40].

To investigate the contribution of proteasome impairment in genetic prion diseases, we measured the UPS efficiency in Tg(PG14) mice. As these mice age they progressively accumulate an oligomeric form of mutant PrP in the CNS [62], and develop massive apoptotic degeneration of cerebellar granule neurons [9]. This might reflect a gradual loss of the efficiency of the proteasomal degradation system due to build-up of toxic PrP aggregates in the cytosol. Analysis of UPS activity *in vivo* using the Ub^G76V^-GFP reporter did not find specific alterations at any stage of the Tg(PG14) disease. Neither was any loss of UPS efficiency found in neurons expressing D177N PrP, using an innovative fluorogenic peptide for monitoring proteasomal proteolytic activity in live cells. Thus neurodegeneration in the transgenic models is not associated with perturbation of the cytosolic proteasomes, in line with evidence that mutant PrP is not diverted to ERAD in neuronal cells [11], [12]. Finally, there was no loss of proteasome function when PrP was forced to aggregate in the cytosol by a drug that inhibits co-translational translocation. This, and the observation that untranslocated PrP caused no toxicity to neurons [12], [42], strongly argues against the idea that neuronal death in prion diseases is due to cytosolic PrP accumulation.

UPS function was reduced in prion-infected mice [21], [63], but only at an advanced stage of disease and in brain regions with the most intense neuropathology, suggesting that proteasome impairment is a secondary event rather than a primary pathogenic trigger. Moreover, proteasome activity was not reduced in scrapie-infected sheep – in fact, it was higher than in uninfected animals – indicating that low UPS function is not an obligatory correlate of prion pathology [64].

The results here indicate that PG14 and D177N mutants do not activate ER stress-mediated pro-apoptotic mechanisms, nor do they inhibit the proteasomal degradation pathway. Then, how might these mutants damage neurons? Although delayed in their maturation in the ER, PG14 and D177N PrPs eventually reach the cell surface [11]. Molecules that reach the surface become aggregated and PK-resistant [55], and might damage cells by altering membrane properties or interacting abnormally with other proteins on the plasma membrane [3], [65]. Alternatively, intracellular aggregation of mutant PrP might interfere with vesicular traffic impairing delivery of essential cargo molecules to synapses. We did in fact find that D177N PrP expression in N2a cells is associated with an alteration of the GDI/Rab11 pathway governing post-Golgi vesicular traffic [66]. Finally, PG14 and D177N PrP misfolding may affect calcium homeostasis ([67] and A. Senatore and R.C. unpublished observations) with possible consequences on intracellular signaling and neurotransmission.

In conclusion our analyses do not support a role for ER stress and/or UPS dysfunction in prion diseases. Elucidating the proximate cause of neuronal dysfunction and degeneration in these disorders remains an open challenge.

## Materials and Methods

### Mice

Production of transgenic mice expressing wild-type, PG14 and D177N/V128 mouse PrPs with an epitope for the monoclonal antibody 3F4 has been reported [8], [10]. In this study we used transgenic mice of the Tg(WT-E1^+/+^) line, which express ∼4X the endogenous PrP level, referred to throughout the text as Tg(WT), as well as Tg(PG14-A3^+/−^), and Tg(D177N/V128-A21^+/−^) expressing transgenic PrP at ∼1X, referred to as Tg(PG14) and Tg(CJD), respectively. These mice were originally generated on a C57BL/6J X CBA/J hybrid and were then bred with the Zurich I line of *Prnp* ^0/0^ mice [68] with a pure C57BL/6J background (European Mouse Mutant Archive, Monterotondo, Rome, Italy; EM:01723). The Ub^G76V^-GFP1 and Ub^G76V^-GFP2 mouse lines expressing the transgene from a chicken β-actin promoter with a cytomegalovirus immediate early enhancer [32] were maintained by breeding with C57BL/6J mice.

Double transgenic mice expressing PrP and Ub^G76V^-GFP were generated by breeding Tg(WT^+/+^) or Tg(PG14^+/−^) with Ub^G76V^-GFP mice (of both the Ub^G76V^-GFP1 and Ub^G76V^-GFP2 lines). The offspring was screened by PCR for the presence of the transgenes as described [8], [32]. Tg(PG14^+/−^)/Ub^G76V^-GFP^+/−^ mice were analyzed with equal numbers of age-matched controls. These included Tg(PG14^−/−^)/Ub^G76V^-GFP^+/−^, Tg(PG14^+/−^)/Ub^G76V^-GFP^−/−^, and Tg(WT^+/−^)/Ub^G76V^-GFP^+/−^.

Development of neurological illness in PG14 PrP-expressing mice was scored according to a set of objective criteria [8]. Cerebellar degeneration was analyzed by histology as described [9], [22].

### Ethics Statement

All procedures involving animals were conducted according to European Union (EEC Council Directive 86/609, OJ L 358,1; December 12, 1987) and Italian (D.L. n.116, G.U. suppl. 40, February 18, 1992) laws and policies, and were in accordance with the United States Department of Agriculture Animal Welfare Act and the National Institutes of Health Policy on Humane Care and Use of Laboratory Animals. They were reviewed and approved by the Mario Negri Institute Animal Care and Use Committee that includes *ad hoc* members for ethical issues (18/01-D, 18/01-C). Animal facilities meet international standards and are regularly checked by a certified veterinarian who is responsible for health monitoring, animal welfare supervision, experimental protocols and review of procedures.

### Neuronal Cultures

Primary neuronal cultures were prepared as previously described [69]. Briefly, cerebella were dissected, sliced into ∼1-mm pieces and incubated in Hank's balanced salt solution (HBSS, Gibco) containing 0.3 mg/ml trypsin (Sigma) at 37°C for 15 min. Trypsin inhibitor (Sigma) was added to a final concentration of 0.5 mg/ml and the tissue was mechanically dissociated by passing through a flame-polished Pasteur pipette. Cells were plated at 350–400,000 cells/cm^2^ on poly-L-lysine (0.1 mg/ml)-coated plates. Cells were maintained in Basal Medium Eagle (Gibco) supplemented with 10% dialyzed fetal bovine serum (FBS, Sigma), penicillin/streptomycin and KCl 25 mM, at 37°C in an atmosphere of 5% CO_2_, 95% air.

Hippocampal neurons were prepared from two-day-old animals as described [69]. Brain tissue was sliced into ∼1-mm pieces and incubated in HBSS (Gibco) containing 20 U/ml papain (Sigma) at 34°C for 30 min. Trypsin inhibitor (Sigma) was added to a final concentration of 0.5 mg/ml and the tissue was mechanically dissociated by passing through a flame-polished Pasteur pipette. Cells were plated at 150–250,000 cells/cm^2^ on poly-D-lysine-coated (25 mg/ml) plates and maintained in Neurobasal Basal Medium (Gibco) supplemented with B27 (Gibco), penicillin/streptomycin and glutamine 2 mM. To reduce the number of non-neuronal cells, aphidicolin (3.3 µg/ml, Sigma) was added to the medium 48 h after plating. Non-neuronal contamination was less than 3%.

### Cell lines

PC12 Tet-on cells expressing the reverse tetracycline-controlled transactivator (rtTA) [26] were grown on poly-L-lysine-coated dishes in Dulbecco's modified Eagle's medium (DMEM, Sigma) supplemented with 10% FBS (Sigma) and 5% horse serum (HS, Sigma) tested for the absence of tetracycline-derived contaminants, at 37°C in a 5% CO_2_ atmosphere. To induce neuronal differentiation cells were seeded on dishes coated with rat tail collagen, and grown in DMEM containing 1% HS, 100 ng/ml NGF (Harlan) and 200 mM 8-(4-chlorophenylthio)adenosine3′:5′-cyclic monophosphate sodium salt (CPT-cAMP, Roche). Cells were used after 7 days, when 80% of cells were differentiated, as judged by neurite outgrowth.

Construction of cDNAs encoding wild-type (M128, WT), M128V, D177N/M128, D177N/V128 or PG14 PrP carrying an epitope tag for the monoclonal antibody 3F4 has been described [12], [70]. Pst I and Not I restriction sites were inserted respectively upstream of the 5′ and downstream of the 3′ ends of the PrP open reading frame by PCR, using Vent polymerase (New England Biolabs) and the following primers: 5′-GACCAGCTGCAGATGGCGAACCTTGGCTACTGGCTG-3′ (sense); 5′-GACCAGGCGGCCGCTCATCCCACGATCAGGAAGATGAG-3′ (antisense). The amplification products were cloned into the Pst I/Not I restriction sites of the bidirectional Tet vector pBI-G (BD Clontech), containing the tetracycline-responsive element (TRE), and their identity was confirmed by sequencing the entire coding region.

PC12 Tet-on cells were plated on 100-mm dishes, left to reach 80% confluence and transfected with 0.8 µg of pBI-G empty or containing the PrP cDNA (WT, M128V, D177N/M128, D177N/V128 or PG14), 8 µg of pTeT-tTS encoding for a transcriptional silencer (tTS) that blocks transcription of genes under control of the TRE in the absence of dox, and 0.1 µg of pTK-Hyg encoding for hygromycin resistance, using Lipofectamine Plus reagent (Invitrogen Inc., Carlsband, California). Three days after transfection, hygromycine (80 µg/ml, Sigma) was added to the culture medium. Resistant clones were isolated four weeks after exposure to hygromycin and tested for PrP expression before and after induction with 1 µg/ml dox for 24 h by immunoblotting with the 3F4 antibody. Only clones with undetectable basal expression of transfected PrP were selected for further experiments. The data presented here were obtained from at least two cloned lines expressing each construct.

HEK (human embryonic kidney)-293 cells (A.T.C.C.; CRL-1573) were grown in a 1∶1 mixture of MEM (minimal essential medium)-α and DMED containing 10% FBS, 2 mM glutamine, non-essential amino acids and penicillin/streptomycin. Cells were transfected with pcDNA3.1 plasmids encoding WT or mutant PrPs [12], or the µ-chain of mouse IgM-H [28], using Lipofectemine™ 2000 (Invitrogen).

### Antibodies

Anti-PrP mouse monoclonal antibody 3F4 [71] was diluted 1∶200 for immunofluorescence and 1∶5,000 for Western blot, and 12B2 [72] 1∶2,500 for Western blot. A rabbit polyclonal antibody against calnexin was used 1∶100. A rabbit polyclonal antibody against GFP (Molecular Probes) was used 1∶2,500. A rabbit polyclonal anti-CHOP/GADD53 antibody (Santa Cruz Biotechnology) was used 1∶50 for immunofluorescence and 1∶800 for immunohistochemistry. Anti-KDEL and anti-Grp58/Erp57 mouse monoclonal antibodies (Stressgen) were used 1∶1,000. An anti-pan-actin monoclonal antibody (Chemicon) was used 1∶20,000. A goat polyclonal anti-Grp78/BiP antibody (Santa Cruz Biothecnology) was used 1∶200 for immunohistochemistry. A horseradish peroxidase-conjugated anti-mouse IgM antibody (Santa Cruz Biotechnology) was used 1∶5,000 to detect murine Ig-µ-chains by Western blot.

### XBP1 Splicing

Total RNA was extracted using RNAwiz (Ambion), according to the manufacturer's instructions. RNA samples were reverse-transcribed with MuLV Reverse Transcriptase (Applied Biosystems) by priming with oligo(dT). XBP1 mRNA was amplified with primers flanking the 26b intron (5′-GGAGTGGAGTAAGGCTGGTG and 5′-CCAGAATGCCCAAAAGGATA) and PCR products resolved on 2.5% agarose gels [19].

### Northern Blot

Northern blot analysis was done using the CDP-Star Chemiluminescent Detection System (Amersham Biosciences). 20 µg of total RNA was separated by electrophoresis on 1.25% agarose gel containing 6% formaldehyde and transferred onto a nylon membrane (Hybond-N+, Amersham Biosciences) by capillary elution for 18 h, then immobilized by baking the membrane for 2 h at 80°C. Mouse PrP (wild-type, 3F4 tagged), hamster CHOP, mouse Grp78/BiP and mouse GADPH (glyceraldehyde-3 phosphate-dehydrogenase) cDNAs were used as a template for the preparation of fluorescein-labeled probes using the Gene Images Random-Prime Labeling module (Amersham Biosciences). The RNA blot was rinsed in SSC 2X (sodium chloride sodium citric acid), pre-hybridized for 2 h at 60°C in hybridization buffer (SSC 5X, 5% dextrane sulphate, 0.1% sodium dodecyl sulphate (SDS), 100 µg/ml denatured salmon sperm DNA, and 10% Liquid Block provided in the Gene Images Blot Kit, Amersham Biosciences) and hybridized for 18 h at 60°C with the labeled probe. Excess probe was removed by washing once with SSC 2X, 0.1% SDS for 15 min at room temperature, once with SSC 1X, 0.1% SDS for 15 min at 65°C, and once with SSC 0.5X, 0.1% SDS for 15 min at 65°C. The membrane was pre-incubated with 5% non-fat dried milk (NFDM) in 0.1 M Tris-HCl, pH 9.5, 0.3 M NaCl (blocking buffer) for 2 h at room temperature before incubating with alkaline phosphatase-conjugated anti-fluorescein antibody (1∶5,000 in blocking buffer) for 1 h at room temperature. Unbound antibody was removed by rinsing four times with 0.1 M Tris-HCl, pH 9.5, 0.3 M NaCl, 0.3% Tween-20. The membrane was incubated for 5 min with the CDP-Star substrate (Amersham Biosciences) and the chemiluminescent signal visualized with a Biorad XRS image scanner or Hyperfilm-MP films (Amersham Biosciences).

### Biochemical Analysis

To test the detergent insolubility of PrP, cells were lysed in 10 mM Tris pH 7.5, 100 mM NaCl, 0.5% sodium deoxycholate and 0.5% Nonidet P-40 containing protease inhibitors (1 µg/ml pepstatin and leupeptin, 0.5 mM phenylmethylsulfonyl fluoride (PMSF), and 2 mM ethylenediaminetetraacetic acid). Lysates corresponding to 300 µg of protein were centrifuged at 186,000 x *g* for 40 min in a Beckman Optima Max-E ultracentrifuge. Proteins in the pellet and supernatant were separated by SDS-polyacrylamide gel electrophoresis and electro-transferred onto polyvinylidene fluoride membranes (Immobilon P, Millipore). Membranes were incubated first with 5% NFDM in 100 mM Tris pH 7.5, 150 mM NaCl and 0.1% Tween 20 (TTBS), then with anti-PrP antibodies overnight at 4°C or for 1 h at room temperature, rinsed three times with TTBS and incubated for 1 h at room temperature with horseradish peroxidase-conjugated secondary antibody (diluted 1∶5,000; Santa Cruz). Signals were revealed using enhanced chemiluminescence (Amersham Biosciences), and visualized by a Biorad XRS image scanner. Quantitative densitometry of protein bands was done using Quantity One software (Biorad).

To test PK resistance 300 µg of protein extracts were digested with 0.25–0.5 µg/ml of PK for 30 min at 37°C. Digestion was terminated by adding PMSF to a final concentration of 5 mM. Proteins were methanol-precipitated and PrP was analyzed by Western blot using the 3F4 antibody.

### Immunofluorescence

PC12 cells were seeded on poly-L-lysine-coated glass coverslips in 24-well plates at 50% confluence and induced to express PrP by treating them with 1 µg/ml dox for 24 h. Alternatively, cells were differentiated with NGF before proceeding with immunofluorescence. For surface staining of PrP, living cells were incubated with anti-PrP 3F4 antibody (1∶500 in OptiMEM) for 1 h at 4°C, washed with ice-cold PBS and fixed in 4% paraformaldehyde (PFA) for 1 h at 4°C. After washing with PBS, cells were incubated in blocking solution (0.5% BSA and 50 mM NH_4_Cl in PBS) for 1 h at room temperature, then with Alexa 488(green)-conjugated goat anti-mouse IgG (Molecular Probes) (1∶500 in blocking solution). After 1 h incubation at room temperature cells were rinsed and mounted in FluorSave (Calbiochem Corporation, San Diego, California). To visualize intracellular PrP and other intracellular antigens, cells were washed with PBS and fixed in 4% PFA for 1 h at 4°C. Cells were then rinsed, permeabilized with 0.1% Triton X-100 for 2 min and incubated for 30 min at room temperature in blocking solution, then with the primary antibody (in blocking solution) for 1 h. Cells were rinsed in 0.1% Tween-20/PBS and incubated with Alexa 488(green)- or 546(red)-conjugated secondary antibody before mounting. For co-localisation experiments, anti-PrP antibody was incubated, followed by the corresponding secondary antibody, before proceeding with organelle markers staining. Cells were analyzed with an Olympus FV500 laser confocal scanning system.

### Immunohistochemistry

For CHOP/GADD153 and Grp78/Bip immunostaining, sections from paraffin-embedded, 4% PFA-fixed tissue were collected on collagen-coated slides and stained using an automated immunostaining system (Leica). Endogenous peroxidase activity was blocked with 0.3% H_2_O_2_ for 20 min at room temperature. Primary antibodies were applied to slides for 30 min at room temperature, then incubated with horseradish peroxidase-labeled anti-rabbit or anti-goat Envision polymers (Dako) for 30 minutes. The reaction product was developed using 3,3-diaminobenzidine.

For GFP immunostaining, anesthetized mice were intracardially perfused with 4% PFA; their brains were removed, post-fixed and frozen at -80°C after cryoprotection. Brain sections were cut using a Leica cryostat and incubated for 1 h at RT with 10% normal goat serum (NGS), 0.1% Triton X-100 in phosphate-buffered saline (PBS) then overnight at 4°C with the anti-GFP antibody. After incubation with an anti-rabbit IgG biotinylated antibody (Vector Laboratories) immunostaining was developed using the avidin-biotin-peroxidase complex (Vector Laboratories) and diaminobenzidine (Sigma). Images were acquired using an Olympus image analyzer and the Cell-R software.

### Proteasome activity in single cells

Proteasome activity was assessed in living neuronal cells using the TED peptide, as described [36]. Briefly, neurons grown in 15-µm slide 8-well chamber dishes (Ibidi) were incubated with 40 µM TED in Krebs Ringer Hepes (KRH) buffer (125 mM NaCl, 5 mM KCl, 1.2 mM MgSO_4_, 2 mM CaCl_2_, 10 mM glucose, 25 mM Hepes, pH 7.4) for 20 min. The TED solution was replaced with fresh culture medium, and images were collected and analyzed with a Cell^R^ imaging station (Olympus) coupled to an inverted microscope (IX 81, Olympus) equipped with an incubator to maintain constant temperature (37°C) and CO_2_ (5%). The EDANS fluorescent signal was acquired with a high-resolution camera (ORCA) equipped with a 340-nm excitation filter (D340xv2 Chroma), a 400-nm dichroic mirror (400DCLP, Chroma) and an emission filter with a range of 510±40 nm (D510/40 m, Chroma). Images were acquired after 20 min of incubation, and ten frames were randomly sampled for a minimum of 1000 cells. EDANS-positive cells, identified after removing background, were counted and compared to the total cell number counted by bright-field using the ImageJ 1.43 software (http://rsbweb.nih.gov/ij/).

## Supporting Information

Figure S1 **Mutant PrP expression does not trigger ER stress response in HEK-293 cells.** (A, B) HEK-293 cells were transfected with the empty pcDNA3 plasmid (Vec) or with plasmids encoding WT, D177N/M128, D177N/V128, PG14 PrP, or the heavy-chain of mouse IgM (Ig-µ) and lysed after 24 h. Lysates corresponding to 30 µg of proteins were analyzed by Western blot using the 3F4 antibody. Molecular mass markers are in kDa. (C) Total RNA was extracted from transfected HEK-293 cells, and XBP1 splicing was analyzed by RT-PCR. Each product of amplification was separated on 2.5% agarose gel. Spliced forms were detected only in cells expressing the µ-chain or treated with tunicamycin (Tm). Size markers are given in base-pairs.
(TIF)

## References

[1] Prusiner SB (1998). Prions.. Proc Natl Acad Sci U S A.

[2] Collinge J (2001). Prion diseases of humans and animals: their causes and molecular basis.. Annu Rev Neurosci.

[3] Chiesa R, Harris DA (2001). Prion diseases: what is the neurotoxic molecule?. Neurobiol Dis.

[4] Westergard L, Christensen HM, Harris DA (2007). The cellular prion protein (PrP^C^): its physiological function and role in disease.. Biochim Biophys Acta.

[5] Campana V, Sarnataro D, Zurzolo C (2005). The highways and byways of prion protein trafficking.. Trends Cell Biol.

[6] Young K, Piccardo P, Dlouhy S, Bugiani O, Tagliavini F, Harris DA (1999). The Human Genetic Prion Diseases.. Prions: Molecular and Cellular Biology: Horizon Scientific Press.

[7] Mead S (2006). Prion disease genetics.. Eur J Hum Genet.

[8] Chiesa R, Piccardo P, Ghetti B, Harris DA (1998). Neurological illness in transgenic mice expressing a prion protein with an insertional mutation.. Neuron.

[9] Chiesa R, Drisaldi B, Quaglio E, Migheli A, Piccardo P (2000). Accumulation of protease-resistant prion protein (PrP) and apoptosis of cerebellar granule cells in transgenic mice expressing a PrP insertional mutation.. Proc Natl Acad Sci U S A.

[10] Dossena S, Imeri L, Mangieri M, Garofoli A, Ferrari L (2008). Mutant prion protein expression causes motor and memory deficits and abnormal sleep patterns in a transgenic mouse model.. Neuron.

[11] Drisaldi B, Stewart RS, Adles C, Stewart LR, Quaglio E (2003). Mutant PrP is delayed in its exit from the endoplasmic reticulum, but neither wild-type nor mutant PrP undergoes retrotranslocation prior to proteasomal degradation.. J Biol Chem.

[12] Fioriti L, Dossena S, Stewart LR, Stewart RS, Harris DA (2005). Cytosolic prion protein (PrP) is not toxic in N2a cells and primary neurons expressing pathogenic PrP mutations.. J Biol Chem.

[13] Anelli T, Sitia R (2008). Protein quality control in the early secretory pathway.. EMBO J.

[14] Zhang K, Kaufman RJ (2006). The unfolded protein response: a stress signaling pathway critical for health and disease.. Neurology.

[15] Hetz C, Glimcher LH (2009). Fine-tuning of the unfolded protein response: Assembling the IRE1alpha interactome.. Mol Cell.

[16] Yoshida H, Matsui T, Yamamoto A, Okada T, Mori K (2001). XBP1 mRNA is induced by ATF6 and spliced by IRE1 in response to ER stress to produce a highly active transcription factor.. Cell.

[17] Haze K, Yoshida H, Yanagi H, Yura T, Mori K (1999). Mammalian transcription factor ATF6 is synthesized as a transmembrane protein and activated by proteolysis in response to endoplasmic reticulum stress.. Mol Biol Cell.

[18] Lee K, Tirasophon W, Shen X, Michalak M, Prywes R (2002). IRE1-mediated unconventional mRNA splicing and S2P-mediated ATF6 cleavage merge to regulate XBP1 in signaling the unfolded protein response.. Genes Dev.

[19] Orsi A, Fioriti L, Chiesa R, Sitia R (2006). Conditions of endoplasmic reticulum stress favor the accumulation of cytosolic prion protein.. J Biol Chem.

[20] Kang SW, Rane NS, Kim SJ, Garrison JL, Taunton J (2006). Substrate-specific translocational attenuation during ER stress defines a pre-emptive quality control pathway.. Cell.

[21] Kristiansen M, Deriziotis P, Dimcheff DE, Jackson GS, Ovaa H (2007). Disease-associated prion protein oligomers inhibit the 26S proteasome.. Mol Cell.

[22] Chiesa R, Piccardo P, Dossena S, Nowoslawski L, Roth KA (2005). Bax deletion prevents neuronal loss but not neurological symptoms in a transgenic model of inherited prion disease.. Proc Natl Acad Sci U S A.

[23] Xing X, Lai M, Wang Y, Xu E, Huang Q (2006). Overexpression of glucose-regulated protein 78 in colon cancer.. Clin Chim Acta.

[24] Rask K, Thorn M, Ponten F, Kraaz W, Sundfeldt K (2000). Increased expression of the transcription factors CCAAT-enhancer binding protein-beta (C/EBBeta) and C/EBzeta (CHOP) correlate with invasiveness of human colorectal cancer.. Int J Cancer.

[25] Greene LA, Tischler AS (1976). Establishment of a noradrenergic clonal line of rat adrenal pheochromocytoma cells which respond to nerve growth factor.. Proc Natl Acad Sci U S A.

[26] Jörnvall H, Blokzijl A, ten Dijke P, Ibanez CF (2001). The orphan receptor serine/threonine kinase ALK7 signals arrest of proliferation and morphological differentiation in a neuronal cell line.. J Biol Chem.

[27] Chiesa R, Harris DA (2000). Nerve growth factor-induced differentiation does not alter the biochemical properties of a mutant prion protein expressed in PC12 cells.. J Neurochem.

[28] Ronzoni R, Anelli T, Brunati M, Cortini M, Fagioli C (2010). Pathogenesis of ER storage disorders: modulating Russell body biogenesis by altering proximal and distal quality control.. Traffic.

[29] Cenci S, Mezghrani A, Cascio P, Bianchi G, Cerruti F (2006). Progressively impaired proteasomal capacity during terminal plasma cell differentiation.. EMBO J.

[30] Yedidia Y, Horonchik L, Tzaban S, Yanai A, Taraboulos A (2001). Proteasomes and ubiquitin are involved in the turnover of the wild-type prion protein.. Embo J.

[31] Ma J, Lindquist S (2001). Wild-type PrP and a mutant associated with prion disease are subject to retrograde transport and proteasome degradation.. Proc Natl Acad Sci U S A.

[32] Lindsten K, Menendez-Benito V, Masucci MG, Dantuma NP (2003). A transgenic mouse model of the ubiquitin/proteasome system.. Nat Biotechnol.

[33] Bowman AB, Yoo SY, Dantuma NP, Zoghbi HY (2005). Neuronal dysfunction in a polyglutamine disease model occurs in the absence of ubiquitin-proteasome system impairment and inversely correlates with the degree of nuclear inclusion formation.. Hum Mol Genet.

[34] Maynard CJ, Bottcher C, Ortega Z, Smith R, Florea BI (2009). Accumulation of ubiquitin conjugates in a polyglutamine disease model occurs without global ubiquitin/proteasome system impairment.. Proc Natl Acad Sci U S A.

[35] Cheroni C, Marino M, Tortarolo M, Veglianese P, De Biasi S (2009). Functional alterations of the ubiquitin-proteasome system in motor neurons of a mouse model of familial amyotrophic lateral sclerosis.. Hum Mol Genet.

[36] Urru SA, Veglianese P, De Luigi A, Fumagalli E, Erba E (2010). A new fluorogenic peptide determines proteasome activity in single cells.. J Med Chem.

[37] Menendez-Benito V, Verhoef LG, Masucci MG, Dantuma NP (2005). Endoplasmic reticulum stress compromises the ubiquitin-proteasome system.. Hum Mol Genet.

[38] Rane NS, Kang SW, Chakrabarti O, Feigenbaum L, Hegde RS (2008). Reduced translocation of nascent prion protein during ER stress contributes to neurodegeneration.. Dev Cell.

[39] Ma J, Wollmann R, Lindquist S (2002). Neurotoxicity and neurodegeneration when PrP accumulates in the cytosol.. Science.

[40] Soto C (2008). Endoplasmic reticulum stress, PrP trafficking, and neurodegeneration.. Dev Cell.

[41] Harant H, Lettner N, Hofer L, Oberhauser B, de Vries JE (2006). The translocation inhibitor CAM741 interferes with vascular cell adhesion molecule 1 signal peptide insertion at the translocon.. J Biol Chem.

[42] Restelli E, Fioriti L, Mantovani S, Airaghi S, Forloni G (2010). Cell type-specific neuroprotective activity of untranslocated prion protein.. PLoS One.

[43] Yoo BC, Krapfenbauer K, Cairns N, Belay G, Bajo M (2002). Overexpressed protein disulfide isomerase in brains of patients with sporadic Creutzfeldt-Jakob disease.. Neurosci Lett.

[44] Hetz C, Russelakis-Carneiro M, Maundrell K, Castilla J, Soto C (2003). Caspase-12 and endoplasmic reticulum stress mediate neurotoxicity of pathological prion protein.. Embo J.

[45] Hetz C, Russelakis-Carneiro M, Walchli S, Carboni S, Vial-Knecht E (2005). The disulfide isomerase Grp58 is a protective factor against prion neurotoxicity.. J Neurosci.

[46] Tang Y, Xiang W, Terry L, Kretzschmar HA, Windl O (2010). Transcriptional analysis implicates endoplasmic reticulum stress in bovine spongiform encephalopathy.. PLoS One.

[47] Ferreiro E, Resende R, Costa R, Oliveira CR, Pereira CM (2006). An endoplasmic-reticulum-specific apoptotic pathway is involved in prion and amyloid-beta peptides neurotoxicity.. Neurobiol Dis.

[48] Kocisko DA, Come JH, Priola SA, Chesebro B, Raymond GJ (1994). Cell-free formation of protease-resistant prion protein [see comments].. Nature.

[49] Saborio GP, Permanne B, Soto C (2001). Sensitive detection of pathological prion protein by cyclic amplification of protein misfolding.. Nature.

[50] Caughey B, Raymond GJ (1991). The scrapie-associated form of PrP is made from a cell surface precursor that is both protease- and phospholipase-sensitive.. J Biol Chem.

[51] Borchelt DR, Taraboulos A, Prusiner SB (1992). Evidence for synthesis of scrapie prion proteins in the endocytic pathway.. J Biol Chem.

[52] Marijanovic Z, Caputo A, Campana V, Zurzolo C (2009). Identification of an intracellular site of prion conversion.. PLoS Pathog.

[53] Hetz C, Lee AH, Gonzalez-Romero D, Thielen P, Castilla J (2008). Unfolded protein response transcription factor XBP-1 does not influence prion replication or pathogenesis.. Proc Natl Acad Sci U S A.

[54] Steele AD, Hetz C, Yi CH, Jackson WS, Borkowski AW (2007). Prion pathogenesis is independent of caspase-12.. Prion.

[55] Daude N, Lehmann S, Harris DA (1997). Identification of intermediate steps in the conversion of a mutant prion protein to a scrapie-like form in cultured cells.. Journal of Biological Chemistry.

[56] Tagliavacca L, Anelli T, Fagioli C, Mezghrani A, Ruffato E (2003). The making of a professional secretory cell: architectural and functional changes in the ER during B lymphocyte plasma cell differentiation.. Biol Chem.

[57] Unterberger U, Hoftberger R, Gelpi E, Flicker H, Budka H (2006). Endoplasmic reticulum stress features are prominent in Alzheimer disease but not in prion diseases in vivo.. J Neuropathol Exp Neurol.

[58] Riek R, Wider G, Billeter M, Hornemann S, Glockshuber R (1998). Prion protein NMR structure and familial human spongiform encephalopathies.. Proc Natl Acad Sci U S A.

[59] Apetri AC, Surewicz K, Surewicz WK (2004). The effect of disease-associated mutations on the folding pathway of human prion protein.. J Biol Chem.

[60] Ashok A, Hegde RS (2009). Selective processing and metabolism of disease-causing mutant prion proteins.. PLoS Pathog.

[61] Hetz C, Castilla J, Soto C (2007). Perturbation of endoplasmic reticulum homeostasis facilitates prion replication.. J Biol Chem.

[62] Chiesa R, Piccardo P, Quaglio E, Drisaldi B, Si-Hoe SL (2003). Molecular distinction between pathogenic and infectious properties of the prion protein.. J Virol.

[63] Kang SC, Brown DR, Whiteman M, Li R, Pan T (2004). Prion protein is ubiquitinated after developing protease resistance in the brains of scrapie-infected mice.. J Pathol.

[64] Amici M, Cecarini V, Cuccioloni M, Angeletti M, Barocci S (2010). Interplay between 20S proteasomes and prion proteins in scrapie disease.. J Neurosci Res.

[65] Solomon IH, Schepker JA, Harris DA (2009). Prion neurotoxicity: insights from prion protein mutants.. Curr Issues Mol Biol.

[66] Massignan T, Biasini E, Lauranzano E, Veglianese P, Pignataro M (2010). Mutant prion protein expression is associated with an alteration of the Rab GDP dissociation inhibitor alpha (GDI)/Rab11 pathway.. Mol Cell Proteomics.

[67] Torres M, Castillo K, Armisen R, Stutzin A, Soto C (2011). Prion protein misfolding affects calcium homeostasis and sensitizes cells to endoplasmic reticulum stress.. PLoS One.

[68] Bueler H, Fischer M, Lang Y, Bluethmann H, Lipp HP (1992). Normal development and behaviour of mice lacking the neuronal cell-surface PrP protein.. Nature.

[69] Chiesa R, Fioriti L, Tagliavini F, Salmona M, Forloni G, Lehmann S, Grassi J (2004). Cytotoxicity of PrP peptides.. Techniques in Prion Research.

[70] Lehmann S, Harris DA (1996). Mutant and Infectious Prion Proteins Display Common Biochemical Properties In Cultured Cells.. Journal of Biological Chemistry.

[71] Kascsak RJ, Rubenstein R, Merz PA, Tonna-DeMasi M, Fersko R (1987). Mouse polyclonal and monoclonal antibody to scrapie-associated fibril proteins.. J Virol.

[72] Langeveld JP, Jacobs JG, Erkens JH, Bossers A, van Zijderveld FG (2006). Rapid and discriminatory diagnosis of scrapie and BSE in retro-pharyngeal lymph nodes of sheep.. BMC Vet Res.

