# Supplementary figures and images for: Expression of Mutant or Cytosolic PrP in Transgenic Mice and Cells Is Not Associated with Endoplasmic Reticulum Stress or Proteasome Dysfunction
Source: PLoS One. 2011 Apr 29;6(4):e19339. doi: 10.1371/journal.pone.0019339 (PMC3084828; doi:10.1371/journal.pone.0019339)

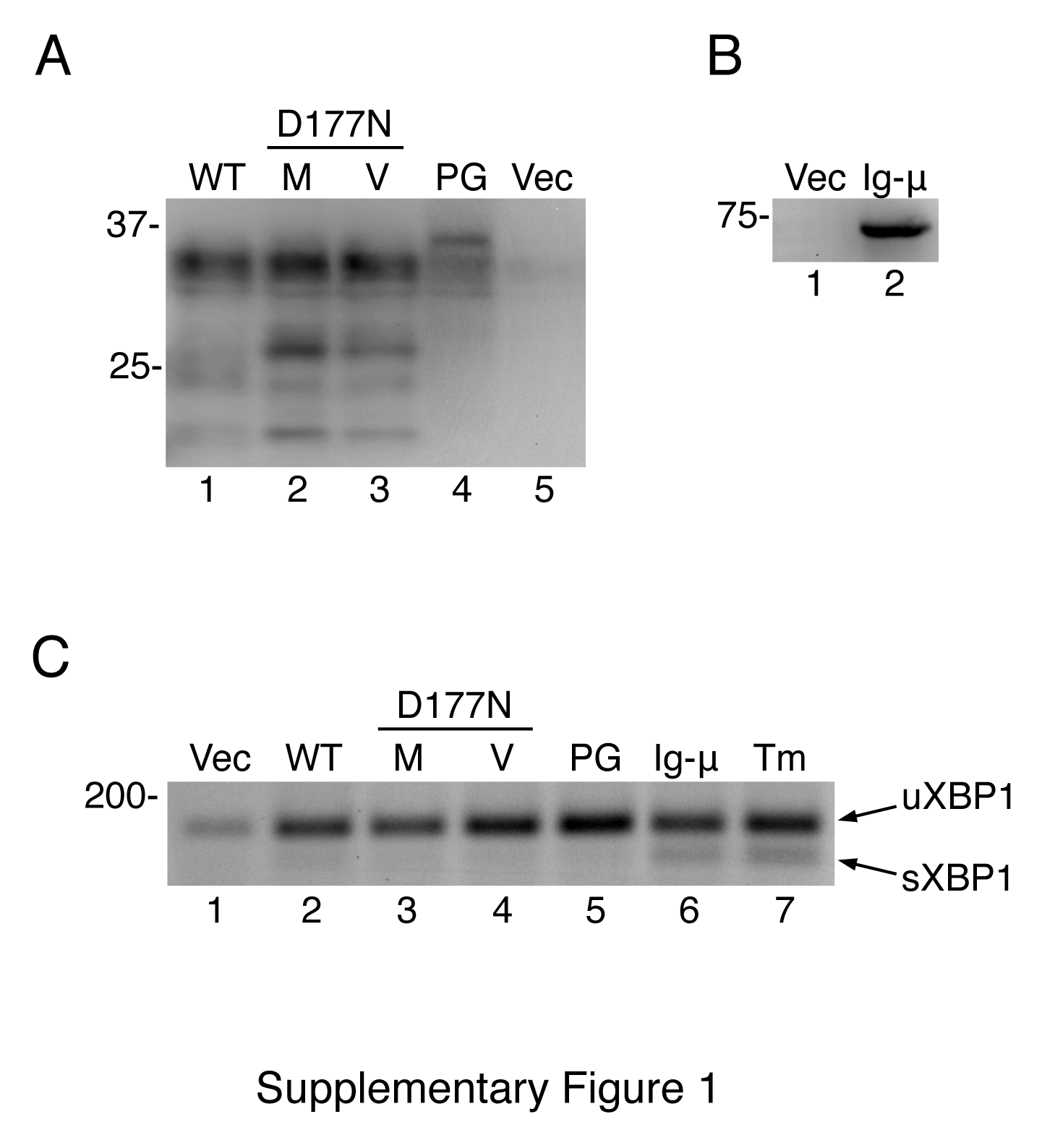

Supplement: Figure S1 — Mutant PrP expression does not trigger ER stress response in HEK-293 cells. (A, B) HEK-293 cells were transfected with the empty pcDNA3 plasmid (Vec) or with plasmids encoding WT, D177N/M128, D177N/V128, PG14 PrP, or the heavy-chain of mouse IgM (Ig-µ) and lysed after 24 h. Lysates corresponding to 30 µg of proteins were analyzed by Western blot using the 3F4 antibody. Molecular mass markers are in kDa. (C) Total RNA was extracted from transfected HEK-293 cells, and XBP1 splicing was analyzed by RT-PCR. Each product of amplification was separated on 2.5% agarose gel. Spliced forms were detected only in cells expressing the µ-chain or treated with tunicamycin (Tm). Size markers are given in base-pairs. (TIF) [file pone.0019339.s001.tif]
